# Melatonin treatment improves human umbilical cord mesenchymal stem cell therapy in a mouse model of type II diabetes mellitus via the PI3K/AKT signaling pathway

**DOI:** 10.1186/s13287-022-02832-0

**Published:** 2022-04-12

**Authors:** Aili Aierken, Balun Li, Peng Liu, Xuedi Cheng, Zheng Kou, Ning Tan, Mengfei Zhang, Shuai Yu, Qiaoyan Shen, Xiaomin Du, Bold Bayar Enkhbaatar, Juqing Zhang, Rui Zhang, Xiaolong Wu, Ruibin Wang, Xin He, Na Li, Sha Peng, Wenwen Jia, Congrong Wang, Jinlian Hua

**Affiliations:** 1 College of Veterinary Medicine, Shaanxi Centre of Stem Cells Engineering and Technology, Northwest A&F University, YanglingShaanxi, 712100 China; 2 Department of Endocrinology and Metabolism, Shanghai Key Laboratory of Diabetes Mellitus, Shanghai Jiao Tong University Affiliated Sixth People’s Hospital, Shanghai, 200233 China; 3 Institute for Regenerative Medicine, National Stem Cell Translational Resource Center, Shanghai East Hospital, School of Life Sciences and Technology, Tongji University, Shanghai, 200092 China; 4 Department of Endocrinology and Metabolism, Shanghai Fourth People’s Hospital, School of Medicine, Tongji University, Shanghai, 200434 China

**Keywords:** Type II diabetes mellitus, Human umbilical cord mesenchymal stem cell (hUC-MSC), Melatonin, PI3K/AKT signaling pathway

## Abstract

**Background:**

Mesenchymal stem cells (MSCs) are promising candidates for tissue regeneration and disease treatment. However, long-term in vitro passaging leads to stemness loss of MSCs, resulting in failure of MSC therapy. This study investigated whether the combination of melatonin and human umbilical cord mesenchymal stem cells (hUC-MSCs) was superior to hUC-MSCs alone in ameliorating high-fat diet and streptozocin (STZ)-induced type II diabetes mellitus (T2DM) in a mouse model.

**Methods:**

Mice were divided into four groups: normal control (NC) group; T2DM group; hUC-MSCs treatment alone (UCMSC) group and pretreatment of hUC-MSCs with melatonin (UCMSC/Mel) group.

**Results:**

RNA sequence analysis showed that certain pathways, including the signaling pathway involved in the regulation of cell proliferation signaling pathway, were regulated by melatonin. The blood glucose levels of the mice in the UCMSC and UCMSC/Mel treatment groups were significantly reduced compared with the T2DM group without treatment (*P* < 0.05). Furthermore, hUC-MSCs enhance the key factor in the activation of the PI3K/Akt pathway in T2DM mouse hepatocytes.

**Conclusion:**

The pretreatment of hUC-MSCs with melatonin partly boosted cell efficiency and thereby alleviated impaired glycemic control and insulin resistance. This study provides a practical strategy to improve the application of hUC-MSCs in diabetes mellitus and cytotherapy.

**Graphical abstract:**

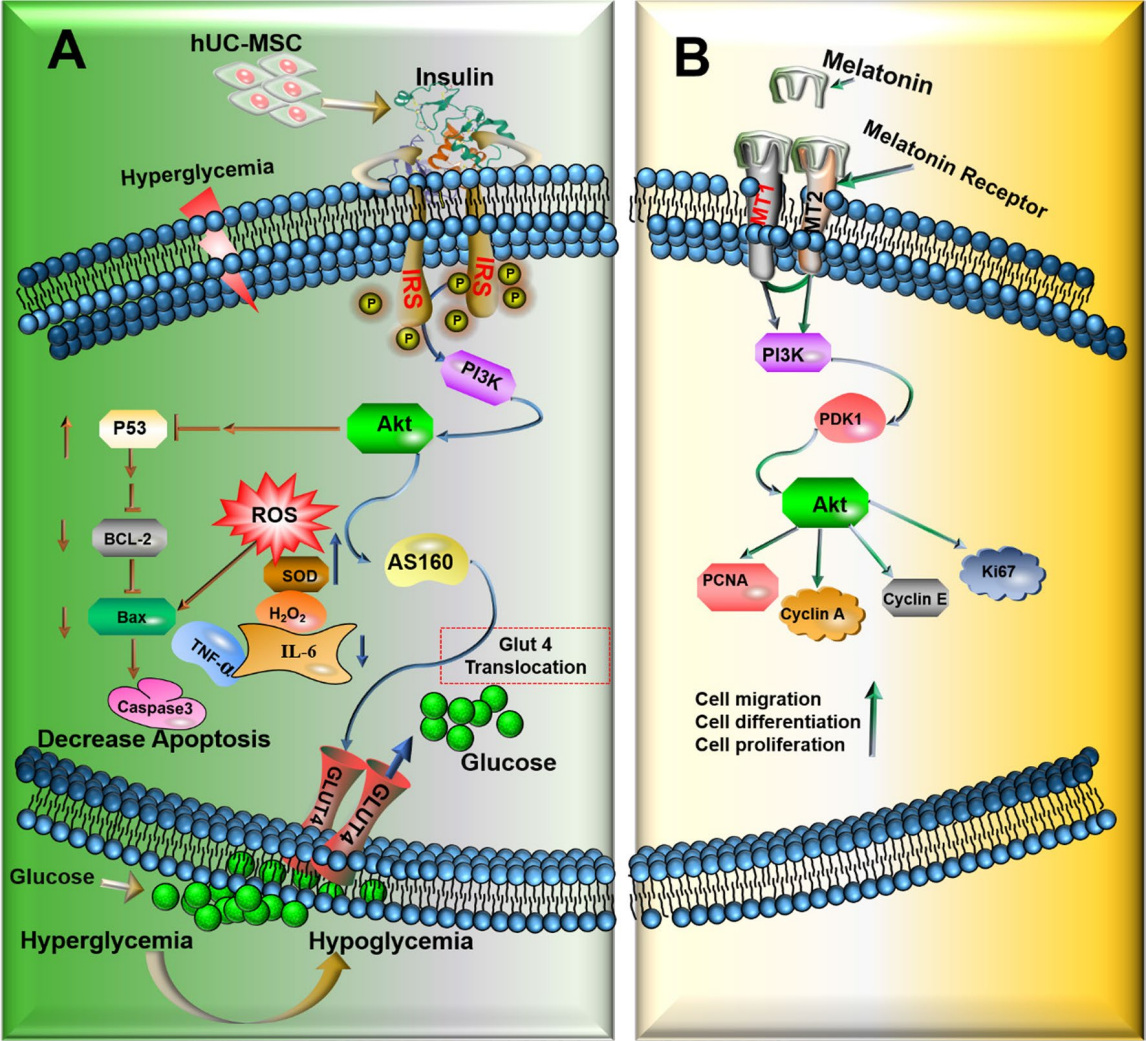

Overview of the PI3K/AKT signaling pathway. (A) Underlying mechanism of UCMSC/Mel inhibition of hyperglycemia and insulin resistance T2DM mice via regulation of PI3K/AKT pathway. hUC-MSCs stimulates glucose uptake and improves insulin action thus should inhibition the clinical signs of T2DM, through activation of the p-PI3K/Akt signaling pathway and then regulates glucose transport through activating AS160. UCMSC/Mel increases p53-dependent expression of BCL2, and inhibit BAX and Capase3 protein activation. Leading to the decrease in apoptosis. (B) Melatonin modulated PI3K/AKT signaling pathway. Melatonin activated PI3K/AKT response pathway through binding to MT1and MT2 receptor. Leading to the increase in hUC-MSCs proliferation, migration and differentiation. → (Direct stimulatory modification); ┴ ( Direct Inhibitory modification); → ┤ (Multistep inhibitory modification); ↑ (Up regulate); ↓ (Down regulate); PI3K (Phosphoinositide 3-Kinase); AKT ( protein kinase B); PDK1 (Phosphoinositide-dependent protein kinase 1); IR, insulin receptor; GLUT4 ( glucose transporter type 4); ROS (reactive oxygen species); BCL-2 (B-cell lymphoma-2); PDK1 (phosphoinositide-dependent kinase 1) BAX (B-cell lymphoma-2-associated X protein); PCNA (Proliferating cell nuclear antigen); Cell cycle-associated proteins (KI67, cyclin A, cyclin E)

**Supplementary Information:**

The online version contains supplementary material available at 10.1186/s13287-022-02832-0.

## Introduction

Diabetes is one of the most common clinical endocrine metabolic diseases, of which more than 90–95% are type II diabetes mellitus (T2DM) [1]. T2DM is a metabolic disease characterized by high blood glucose levels (hyperglycemia) resulting from insulin secretion deficiency, insulin resistance, or the combination of these two factors [2, 3]. Insulin is the key hormone in mammals that plays a prominent role in regulating the blood glucose level and can lower cholesterol and blood glucose levels. In glucose metabolism, insulin first stimulates the insulin receptor and then activates the phosphoinositide 3-kinase (PI3K)/protein kinase B (AKT) signaling pathway [4, 5]. The PI3K/AKT pathway is the essential node of insulin signaling transduction and modulates blood glucose uptake, cell metabolism, cell survival, proliferation, migration, and glycogen synthesis [6, 7]. Therefore, the PI3K/AKT signaling pathway plays an important role in regulating insulin resistance and hyperglycemia in T2DM.

MSCs are pluripotent stem cells that have the capacity to generate progenitors and differentiate into multiple lineages of the mesenchyme [8]. MSC therapy is a new branch of emerging regenerative therapy in pancreas regeneration and beta-cell and tissue repair [9]. The use of MSCs for cytotherapy relies on the capacity of these cells to colonize, the viability of the cell and ability to engraft long-term into the appropriate damaged tissue or target tissue [10]. Some studies have shown that MSCs are a potential cell-based therapy for treating various diseases, such as liver injury, diabetes mellitus and neurodegenerative diseases [11]. However, our previous study showed that long-term in vitro culture and passage of MSCs resulted in continuous changes to the cells. For example, decreased expression levels of specific surface antigens, anomalous morphology, self-renewal potency, and proliferation rate accelerate senescence [12, 13]. This is becoming a vital issue hindering the clinical application of MSC cytotherapy.

Melatonin (N-acetyl-5-methoxytryptamine, Mel) is a natural hormone that is produced and released via the pineal gland and regulates circadian rhythms (24-h internal clock). Additionally, Mel is capable of inhibiting senescence and inflammatory properties [14, 15]. Our previous study showed that Mel significantly regulates MSC differentiation, proliferation, and anti-apoptotic effects, alleviates the loss of Leydig cells in diabetic testes and provides a healthier niche for spermatogonial stem cells [12, 16]. In addition, melatonin pretreatment also increased the survival rate and curative effect of adipose-derived mesenchymal stem cell (ADMSC) transplantation [12, 15]. Due to the possible synergy of melatonin with MSCs, in this study, we aimed to investigate the therapeutic effect of MSCs, either alone or in combination with melatonin, to improve hUC-MSC cell therapy and the hypoglycemic effect of hUC-MSC in a T2DM mouse model.

## Materials and methods

### Animals and treatment

Male Kunming mice (26 ± 5 g) aged six weeks were obtained from the experimental animal center of the DOSSY EXPERIMENTAL ANIMALS CO., LTD. (Xi’an, China). Mice were allowed free access to distilled water and standard food and were housed in a room under conditions of maintained temperature (24–26 °C) and humidity (69–71%) as well as a 12:12 h light–dark cycle. All experimental protocols were carried out according to the guidelines established by the Chinese National Standard GB/T35892-2018 (guidelines for the ethical review of laboratory animal welfare), submitted and previously approved by the Ethics Committee on the Use of Animals of the Northwest A&F University, approval number (NWAFU.No20191230c0600601[176]). All mice were divided into the T2DM group or the normal control (NC) group, which were given a HFD (protein 26%, carbohydrate 26%, and fat 35%) or a normal mouse diet for 6 weeks, respectively. To obtain a T2DM mouse model, mice were intraperitoneally injected with 40 mg/kg STZ continually for 3 days. One week after STZ administration, we performed oral glucose tolerance tests (OGTTs). The mice were fasted overnight (12 h, free access to water), and glucose (2 g kg^−1^) was gavaged orally with 0 min OGTT values ≥ 7.8 mmol L^−1^ or 120 min OGTT values ≥ 11.1 mmol L^−1^ to ensure the establishment of the T2DM mouse model [17]. Changes in mouse body weight were measured every 7 days; food intake and water intake were recorded every day (in week 7 and week 10, respectively).

All mice were separated into four groups with fifteen mice (*n* = 15) per group: NC group; T2DM group; UCMSC group (the established T2DM model mice received a single infusion of 1 × 10^6^ hUC-MSCs suspended in 0.2 ml of 0.9% sodium chloride (NaCl) through the tail vein once a week for 4 consecutive weeks); UCMSC/Mel group (hUC-MSCs were pretreated with 10 μΜ melatonin (Additional file 1: Figure S1) for 7 d before injection into T2DM model mice, 1 × 10^6^ UCMSC/Mel suspended in 0.2 ml of 0.9% NaCl was administered through the tail vein injection once a week for 4 consecutive weeks).

During intraperitoneal insulin tolerance tests (IPITT), mice were fasted for 6 h and allowed free access to water. After human insulin (1 U kg^−1^) was intraperitoneally injected, tail blood glucose levels were measured at 0, 30, 60, 90, and 120 min using a glucometer.

### hUC-MSC were isolated and cultured

Human umbilical cords were provided by women who gave birth at Shanghai East Hospital (Shanghai, China). All the subjects provided informed consent. hUC-MSCs were isolated, cultured, and characterized according to the ethics committee of the National Stem Cell Translational Resource Center. hUC-MSCs were isolated from fresh umbilical cord tissues as previously described. Briefly, umbilical cords were first washed with phosphate-buffered saline (PBS) containing penicillin and streptomycin thrice to remove the blood. The rinsed cords were cut into 1–3-cm-long segments and placed in culture dishes, and cord vessels were pulled away. Then, the cord segments were minced into pieces and floated in α-MEM (Gibco, C12571500BT, China) supplemented with 5% Ultra GRo-Advanced (Helios Bioscience, HPCFDCGL50, USA), 100 U/mL penicillin and 100 μg/mL streptomycin. The cord pieces were incubated at 37 °C with 5% CO_2_ in a humidified environment and left undisturbed for 72 h, after which fresh complete medium was added. Subsequently, half of the medium was replaced every 3 days, and the cord tissue was removed after colonies of fibroblast-like cells appeared. Cells were trypsinized and passaged until they reached 80–90% confluence. Early-passage hUC-MSCs (passages 2–5) were used for the follow-up experiments [12, 18].

### Biochemical sampling and analysis

At the end of the experiment, the mice were fasted overnight (12 h). The mouse blood samples were collected and centrifuged for 10 min at 3500 rpm at room temperature to obtain blood serum. Serum liver enzymes included aspartate aminotransferase (AST); alanine aminotransferase (ALT); serum lipid levels included triglyceride (TG) and total cholesterol (TC); serum oxidative levels included hydrogen peroxide (H_2_O_2_) and superoxide dismutase (SOD) (Jiancheng Institute of Biotechnology, Nanjing, China). Serum insulin levels were measured by mouse insulin enzyme-linked immunosorbent assay (ELISA, Hufeng Biotechnology, China) kits according to the protocol supplied by the manufacturer [17, 19].

### RNA sequencing

The cells were separated into the UCMSC group and UCMSC/Mel (hUC-MSCs were pretreated with melatonin) group. Melatonin at a concentration of 10 μΜ was used to stimulate hUC-MSCs for 24 h. Information on the potential target genes was subjected to Gene Ontology (GO) enrichment and Kyoto Encyclopedia of Genes and Genomes (KEGG) pathway annotation analysis using the Database for Annotation, and the pathways with a *P* value of < 0.05 were considered reliable.

### PCR analysis

The mRNA levels of the hUC-MSC melatonin receptor markers melatonin receptor 1 (MT1) and melatonin receptor 2 (MT2) were analyzed via PCR. Total mRNA was extracted using the QIAGEN RNeasy mini-Kit (Cat: 74104). RNA was converted into cDNA by reverse transcription. GAPDH was used as internal reference. The following primers were used: GAPDH, forward: AAATTCCATGGCACCGTCAA; reverse: TTCACACCCATGACGAACAT; MT1, forward: GTCTATCGGGTCCAAGGTGG; reverse: TGTCGTCGTTCTTGAGGGAGA; MT2, forward: GTTACGGTCTGAGTGGTTCTT; reverse: CGGATGGTGGCTTAGATGGC.

### Labeling hUC-MSCs with PKH26

hUC-MSCs were stained with a PKH26 red fluorescent cell linker kit (Sigma-Aldrich, MO, USA) and a PKH26 kit for general cell membrane labeling (Additional file 1: Figure S2), which are useful for in vivo cell tracking. The cells were washed in serum-free buffer 2 times prior to resuspension in diluent C for labeling, and the staining was stopped after 3 min by adding serum. After injection of PKH26-labeled hUC-MSCs, red fluorescence represents the number of homing hUC-MSCs. Then, frozen sections of lung, liver, testes, pancreas, heart, kidney, and spleen were cut into 5–8-μm sections at 24 h and 7 days following injection. Three-dimensional reconstruction analysis was observed under a fluorescence microscope [12].

### Histological analysis

For each group of mice, the liver, pancreas, and white adipose tissue were fixed overnight in 4% neutral formalin solution before embedding in paraffin. Tissues were cut into 5-μm-thick sections and stained with hematoxylin and eosin (H&E) and Periodic Acid-Schiff (PAS) [19].

### Immunofluorescent and immunohistochemical analysis

The expression levels of MT1 (1:200, rabbit IgG, Bioss, China), MT2 (1:200, rabbit IgG, Bioss, China), TNF-α (1:100, Immunoway Biotechnology), IL-6 (1:100, Immunoway Biotechnology), and glucose transporter type 4 (GLUT4, 1: 200, Immunoway Biotechnology) were detected by immunofluorescent and immunohistochemical analysis as previously described [19].

### Western blot analysis

Protein was extracted from hUC-MSC samples or liver tissue using radioimmunoprecipitation assay (RIPA) lysis buffer. The primary antibodies were insulin receptor substrate 1 (IRS-1), phosphoinositide 3-kinase (PI3K, 1:1200, Immunoway Biotechnology), protein kinase B (AKT, 1:1200, Immunoway Biotechnology), phosphoinositide-dependent protein kinase 1(PDK1, 1:1200, Immunoway Biotechnology), glucose transporter type 4 (GLUT4, 1:1200, Immunoway Biotechnology), B-cell lymphoma-2-associated X (BAX, 1:1200, Immunoway Biotechnology), proliferating cell nuclear antigen (PCNA, 1:1200, Immunoway Biotechnology), B-cell lymphoma-2 (BCL-2), cell cycle-associated proteins (Ki-67, cyclin A, cyclin E, 1:1200, Immunoway Biotechnology), A 160 kDa substrate of the Akt Ser/Thr kinase (AS160, 1:1200, Immunoway Biotechnology), cysteine protease protein (Caspase3, 1:1200, Immunoway Biotechnology) glyceraldehyde phosphate dehydrogenase (GAPDH, 1:3000), and β-actin (1:3000). The secondary antibodies conjugated to horseradish peroxidase (1:3000, Immunoway Biotechnology). The normalization of other proteins was performed using β-actin and GAPDH western blot assays [19].

### Statistical analysis

Statistical analyses were performed with SPSS version 19.0 (IBM Corporation, Chicago, USA) software, all the experimental data were shown as mean ± SD, and the results were one-way analysis of variance (ANOVA). A *P* values < 0.05 indicated the statistical significance observed in the comparison.

## Results

### hUC-MSC transplantation ameliorated hyperglycemia in T2DM model mice

**Fig. 1:**
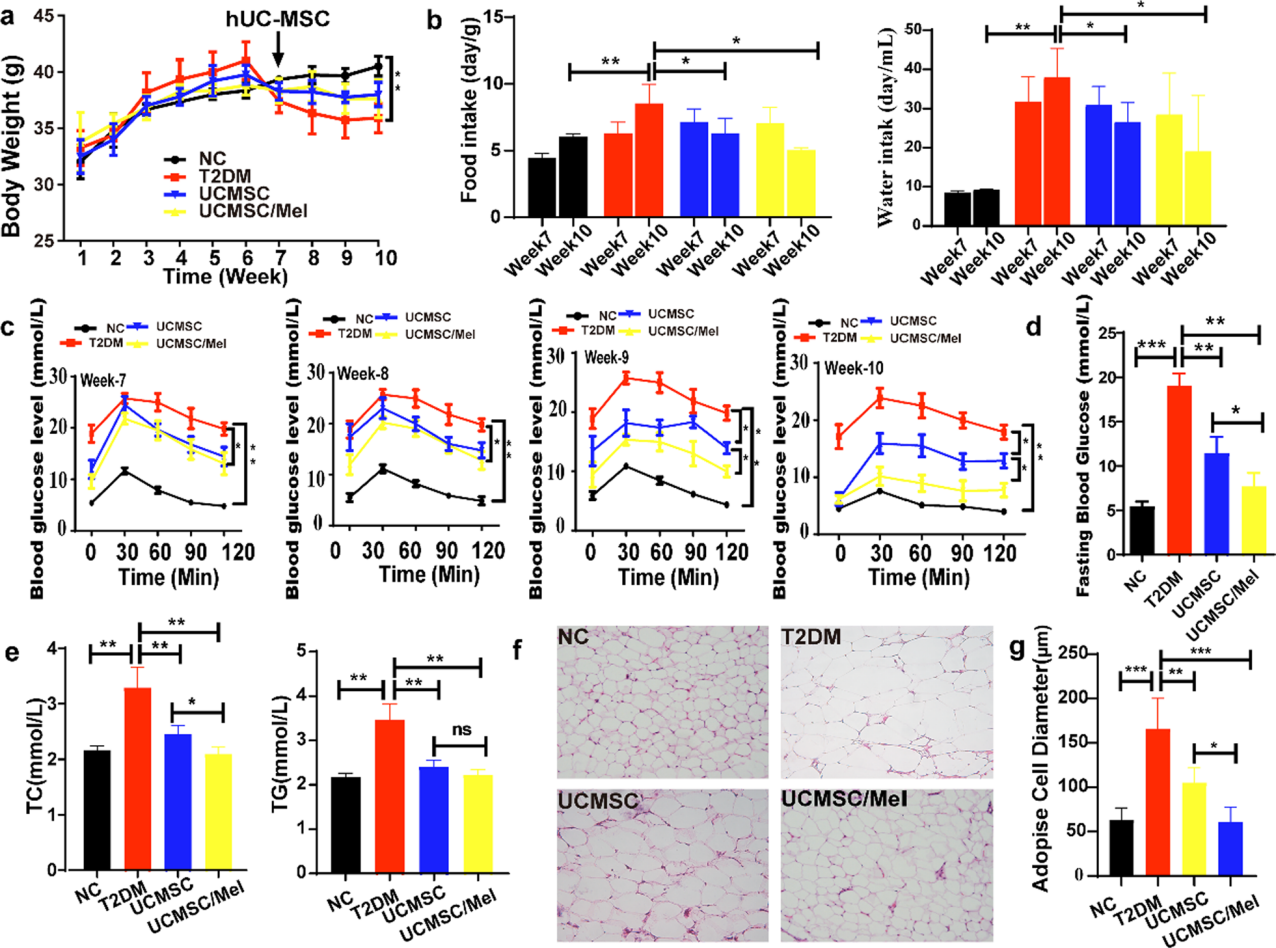
UCMSC infusion improved glucose homeostasis and alleviated dyslipidemia in T2DM mice. **a** Body weight. **b** Food and water intake level. **c** Oral glucose tolerance test. **d** Fasting blood glucose level. **e** Serum total cholesterol (TC) and serum triglyceride (TG) levels among the different groups. **f** Mesenteric white adipose tissue (WAT) H&E staining (magnification ×200). **g** Quantitative analysis of the WAT cell H&E staining results. NC (normal control); T2DM (Type II diabetic mellitus); UCMSC (hUC-MSCs); Mel (melatonin); ns (no significant). Data are mean ± SD of (*n* = 15); ^*^P < 0.05; ^**^P < 0.01; ^***^P < 0.001

During the study period, body weight during the growth phase of all the mice (Fig. 1a) and the changes in body weight suggested that UCMSC infusion had some effect on T2DM mouse weight gain (Fig. 1a). After four weeks of infusion with UCMSCs and UCMSCs/Mel, their food and water intake were significantly lower than those of the T2DM mice. These results suggest that hUC-MSCs/Mel could balance excessive food and water intake in T2DM mice (Fig. 1b, *P* < 0.05). UCMSC- and UCMSC/Mel-treated T2DM mice showed significant moderate decreases in OGTT and FBG (fasting blood glucose levels) (Fig. 1c, d). OGTT levels in the UCMSC and UCMSC/Mel infusion groups significantly decreased at weeks 9 and 10 compared with the T2DM group (*P* < 0.05), and the UCMSC/Mel group revealed significant efficiency compared with the UCMSC group, which indicated that UCMSC/Mel could significantly alleviate the OGTT levels of T2DM mice (Fig. 1c, *P* < 0.05). The FBG level of mice in the NC group was 5.45 ± 0.39 mmol/L, whereas that FBG level of mice in the T2DM group was 19.05 ± 1.9 mmol/L. The FBG levels in the UCMSC and UCMSC/Mel treatment groups were markedly decreased by 11.45 ± 0.87 and 9.4 ± 2.87 mmol/L, respectively, compared with those in the T2DM group (Fig. 1d, *P* < 0.05). The results demonstrate that UCMSC infusion had a positive effect on ameliorating blood glucose levels in T2DM mice.

The effects of hUC-MSCs on the serum lipid levels were investigated in the present study (Fig. 1e). After four weeks of infusion with UCMSCs and UCMSC/Mel, the blood levels of TC and TG in T2DM mice improved significantly. TC and TG levels in the UC-MSC/Mel group were significantly lower than those in the T2DM group. Adipocytes are round or polygonal, and the centers of the cells contain a large lipid droplet; the cytoplasm and nucleus are located at the periphery of the cell. HE staining of adipose tissue was performed (Fig. 1f). The adipocytes of the NC group were neatly arranged and uniform in size, while the entire volume of the adipocytes in the T2DM group was significantly large. The size of the cells was different, and even large cells were observed compared with the NC group (Fig. 1f, *P* < 0.05). The pathological damage was improved to some extent in the UCMSC group. Despite the presence of large cells in the tissue, they were few in number, and the cells were arranged neatly. The most obvious improvement was observed in the UCMSC/Mel group (Fig. 1g, *P*< 0.05).

### Melatonin activates melatonin receptor 1 and melatonin receptor 2 in hUC-MSCs

**Fig. 2:**
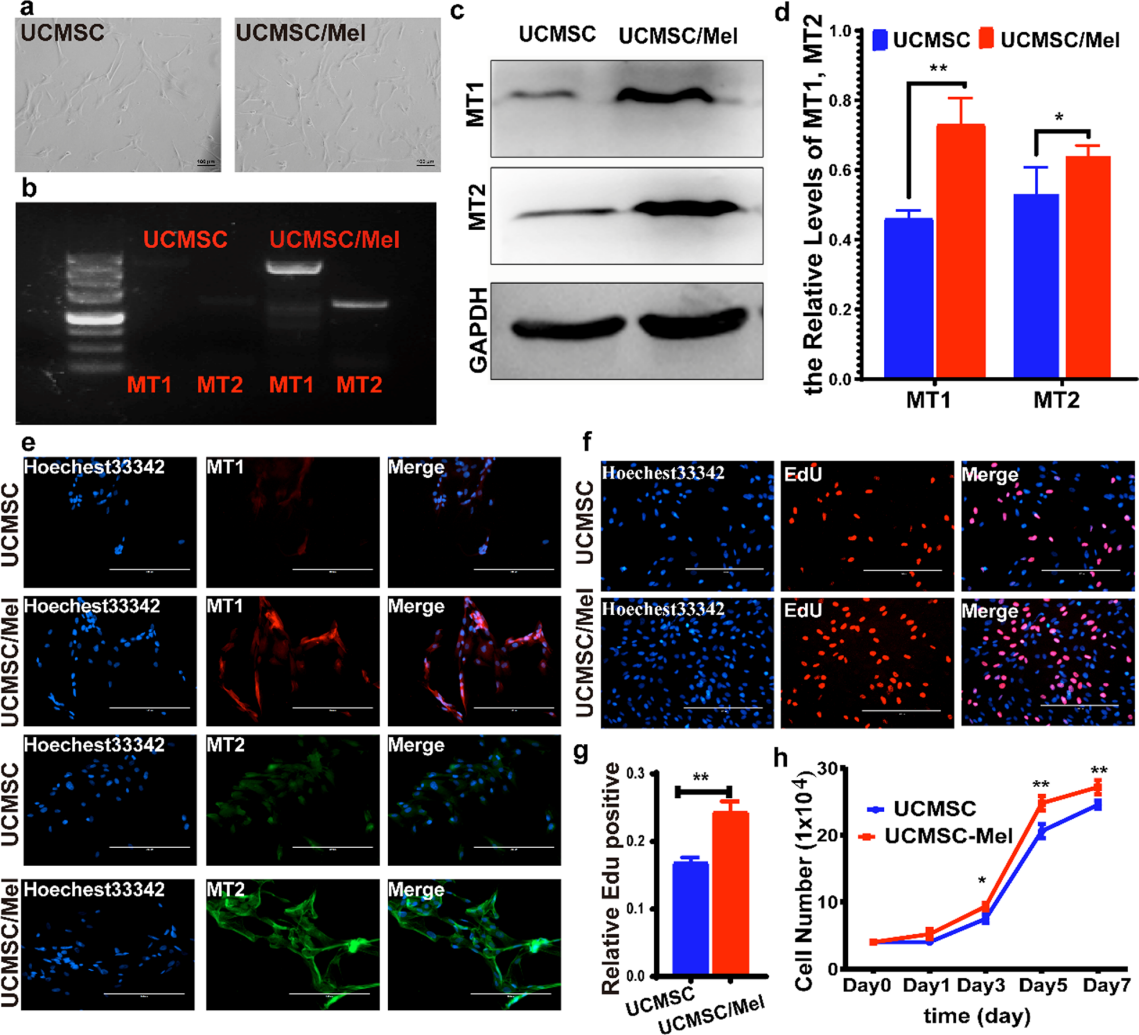
Melatonin upregulates MT1 and MT2 expression. **a** Cell morphology of hUC-MSCs. **b** Semiquantitative RT–PCR analysis of MT1 and MT2 expression in hUC-MSCs treated with melatonin. **c** Western blot analysis of MT1 and MT2 expression levels in hUC-MSCs treated with melatonin. GAPDH was used as a loading control. **d** The relative levels of MT1 and MT2 in the UCMSC and UCMSC/Mel groups. **e** Cell immunofluorescence MT1 and MT2 expression in untreated cells (UCMSCs) and melatonin cocultured hUC-MSCs (UCMSCs/Mel). **f** EdU staining in UCMSCs and UCMSCs/Mel. **g** Cell proliferation rate analyzed by EdU staining. **h** Cell growth curve of untreated cells (UCMSCs) and melatonin-cocultured hUC-MSCs (UCMSCs/Mel). MT1 (melatonin receptor 1); MT2 (melatonin receptor 2); UCMSC (hUC-MSCs); Mel (melatonin). Data are mean ± SD; ^*^P < 0.05; ^**^P < 0.01; ^***^P < 0.001.

To evaluate the importance of melatonin receptors (MT1 and MT2) in facilitating the therapeutic functions of melatonin, we performed immunofluorescence, western blot and semiquantitative PCR analyses of melatonin receptors in hUC-MSCs (UCMSCs) and hUC-MSCs treated with melatonin (UCMSCs/Mel) (Fig. 2). The untreated cells showed weak signals of MT1 and MT2 (Fig. 2b, c and e); however, the fluorescent signals of MT1 and MT2 were highly enriched in UCMSCs cotreated with 10 µm melatonin (Fig. 2b–e). Hence, the quantitative results of the immunofluorescence staining demonstrate that melatonin upregulated MT1 and MT2 expression levels. To examine whether the loss in cell proliferation could be associated with melatonin treatment, we tested the growth of melatonin-cocultured UCMSCs and then analyzed them by using EdU staining and cell proliferation curves. In this study, the Mel treatment group showed increased cell proliferation compared with the control group (Fig. 2f–2h, *P* < 0.05).

### Melatonin upregulates MT1 and MT2 expression through the activation of the PI3K/AKT signaling pathway

**Fig. 3:**
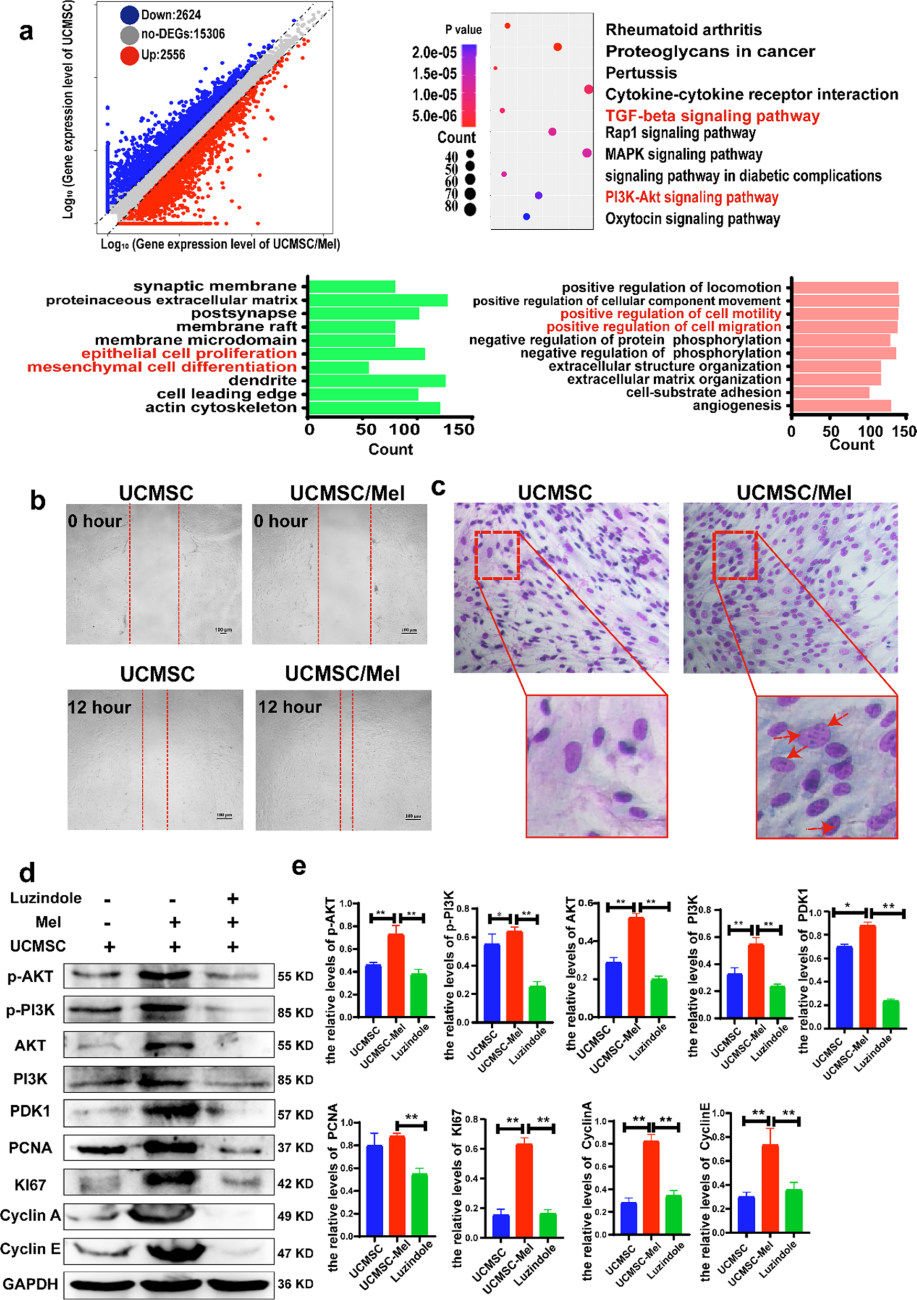
Identification of DEGs in UCMSCs and UCMSCs/Mel. **a** RNA sequence (volcano maps overall distribution of the differential genes; KEGG analysis and GO analysis. **b** Transwell migration assay. **c** Giemsa staining. **d** Western blot analysis of AKT, PI3K, PDK1, PCNA, KI67, Cyclin E and Cyclin A expression levels in hUC-MSCs treated with melatonin and melatonin receptor inhibitor (luzindole). GAPDH was used as a loading control. **e** Quantitative analysis of the western blot results. UCMSC (hUC-MSCs); Mel (melatonin). Data are mean ± SD; ^*^P < 0.05; ^**^P < 0.01; ^***^P < 0.001

We further verified the gene expression changes in UCMSCs after melatonin treatment with RNA sequencing. The data showed that there were 20,486 expressed genes in UCMSCs/Mel cells compared with UCMSCs. Among the 5180 differentially expressed genes (DEGs), 2556 genes were upregulated, 2624 genes were downregulated, and 15,036 genes remained unchanged in UCMSCs/Mel. DEGs were defined as those with expression differences ≥ 2.0. The scatter plot is shown in Fig. 3a, which shows the distribution of differentially expressed genes responsible for cell proliferation, differentiation, the cell cycle, the circadian clock, and cell migration. KI67, PCNA and cyclin D were the significantly upregulated genes, and the absolute multiple changes were 6.8, 16.42, and 20.96, respectively. BAX and Capase3 were the significantly downregulated genes, and the absolute multiple changes were 23 and 15.6, respectively. The scatter plot (Fig. 3a) shows the change in DEG expression between the two groups. There was a significant difference in gene expression between the UCMSC and UCMSC/Mel groups.

The above results show that melatonin activates the melatonin receptors MT1 and MT2 on the surface of UCMSCs (Fig. 1e). To further test whether melatonin could increase the migration and differentiation of UCMSCs, wound healing and Transwell migration assays were conducted. The results demonstrated that 10-μM melatonin treatment effectively promoted wound healing (Fig. 3b), and Giemsa staining indicated that the melatonin treatment group had more nucleolar contrast than the untreated group (Fig. 3c). After treatment with a melatonin inhibitor (luzindole), the expression levels of cell proliferation and cell cycle-related genes were significantly changed (Fig. 3d, e).

### UCMSC/Mel infusion improved insulin resistance and promoted islet recovery in T2DM mice

**Fig. 4:**
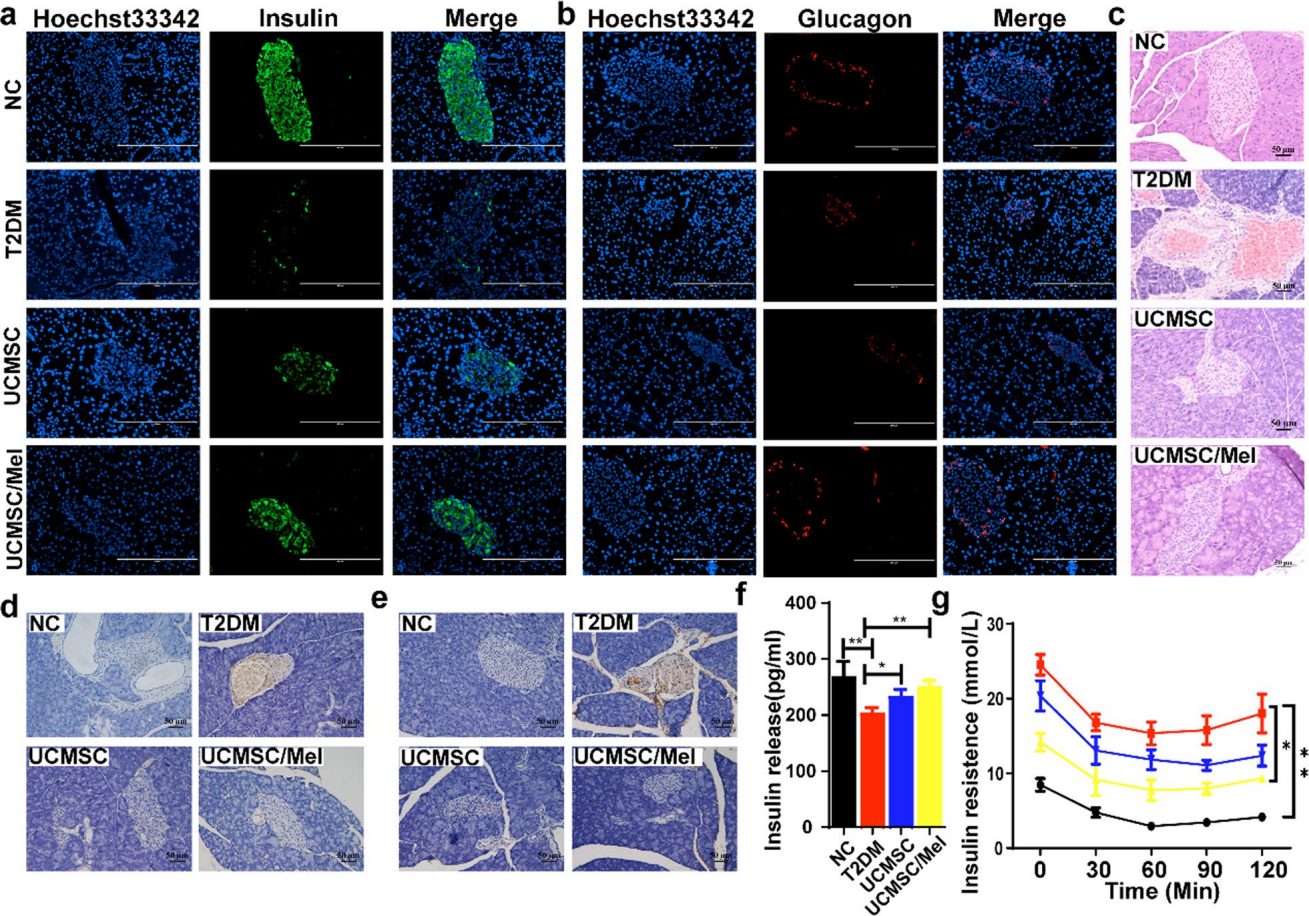
Infusion of UCMSCs promotes restoration of pancreatic islet function in T2DM mice. a Immunofluorescence staining of insulin in pancreatic tissue. **b** Immunofluorescence staining of pancreatic tissue glycogen. **c** H&E staining of pancreas tissue sections. Scale bar 100 µm. **d** Immunohistochemical staining of pancreatic tissue TNF-α. **e** Immunohistochemical staining of pancreatic tissue IL-6. **f** Serum insulin release levels. **g** Insulin resistance levels. NC (normal control); T2DM (Type II diabetic mellitus); UCMSC (hUC-MSCs); Mel (melatonin). Data are mean ± SD of (*n* = 15); ^*^P < 0.05; ^**^P < 0.01; ^***^P < 0.001

Immunofluorescence and immunohistochemical staining were employed to assess the impact of UCMSCs/Mel on the functions of pancreatic islet β cells. Infusion of hUC-MSCs significantly improved the destruction of islets and produced a morphology similar to that of normal islets. Moreover, UCMSC/Mel infusion groups were detected with a stronger response of color anti-insulin and glucagon positive immunostaining compared with the T2DM (Fig. 4a, b). The insulin-producing β cells were mainly located centrally of the pancreatic islet (Fig. 4a), and glucagon-producing cells were located peripherally of the pancreatic islet (Fig. 4b).

HE staining showed that the complete pancreatic structure had clear β cell boundaries and cell nuclei in the NC group. In contrast, the T2DM group demonstrated significant pancreatic islet cell injury with a loose uniform cellular distribution, focal necrosis, and degeneration. The UCMSC and UCMSC/Mel groups showed injury alleviation effects to different degrees. UCMSC/Mel infusion alleviated these pancreatic histopathological alterations and significantly increased islet number and area (Fig. 4c). The expression levels of the proinflammatory cytokines TNF-α and IL-6 were assessed via immunohistochemistry, and the expression levels of TNF-α and IL-6 in the pancreatic islet cells of T2DM mice were abrogated via hUC-MSC infusion in the UCMSC and UCMSC/Mel groups (Fig. 4d, e). After hUC-MSC infusion, the serum insulin level was significantly increased (Fig. 4f, P < 0.01), and there was an obvious decline in insulin resistance (Fig. 4 g, *P* < 0.05).

### Colonization of UCMSCs/Mel in the recipient mouse organs

**Fig. 5:**
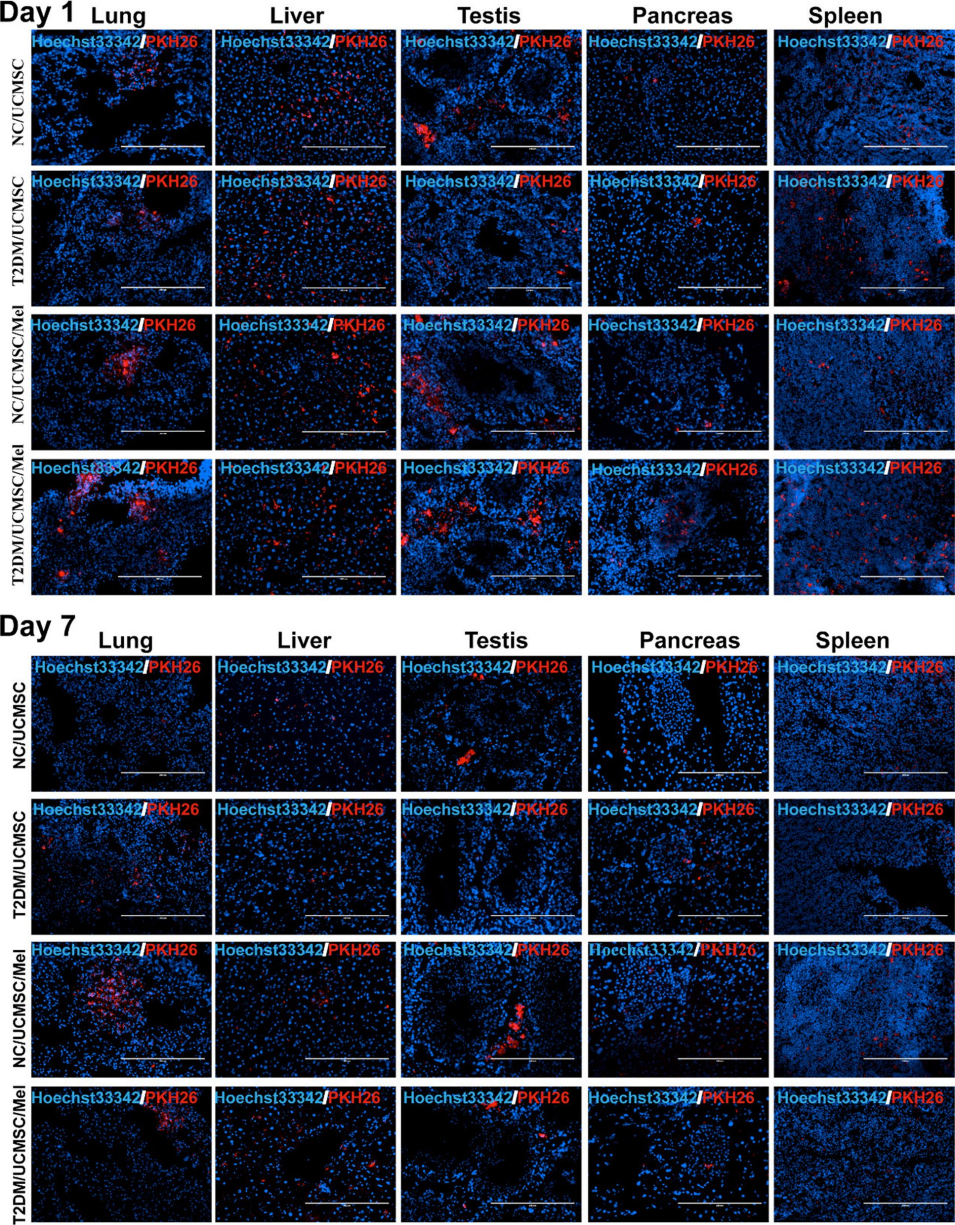
UCMSC homing in the recipient mouse organs. A UCMSCs were PKH26 (red) labeled in advance. After the infusion, recipients were sacrificed on Days 1 and 7, and UCMSC tracking in specific organs (lung, liver, testis, pancreas, and spleen) was evaluated using a confocal laser scanning microscope. NC (normal control); T2DM (Type II diabetic mellitus); UCMSC (hUC-MSCs); Mel (melatonin). Data are mean ± SD of (*n* = 8); ^*^P < 0.05; ^**^P < 0.01; ^***^P < 0.001

To test whether the transplanted cells migrated and were located in the damaged organ, we used PKH26 labeling and transplanted them into T2DM mice. After UCMSC infusion, at Day 1 and Day 7 of UCMSC tracking, some PKH26-labeled UCMSCs could be detected in the lung, liver, testis, pancreas, and spleen (Fig. 5 and Additional file 1: Figure S3) in the NC/UCMSC, NC/UCMSC/Mel, T2DM/UCMSC and T2DM/UCMSC/Mel groups. During the following hours to days, among the organs examined, the liver, lung, testis, and pancreas harbored the largest number of UCMSCs, whereas engraftment of UCMSCs was undetected in the heart and kidney of T2DM mice among the organs examined (Additional file 1: Figure S4). The colonization level of melatonin-treated cells was higher than that of untreated cells.

### Hypoglycemic effects of UCMSCs via regulation of the PI3K/AKT signaling pathway

**Fig. 6:**
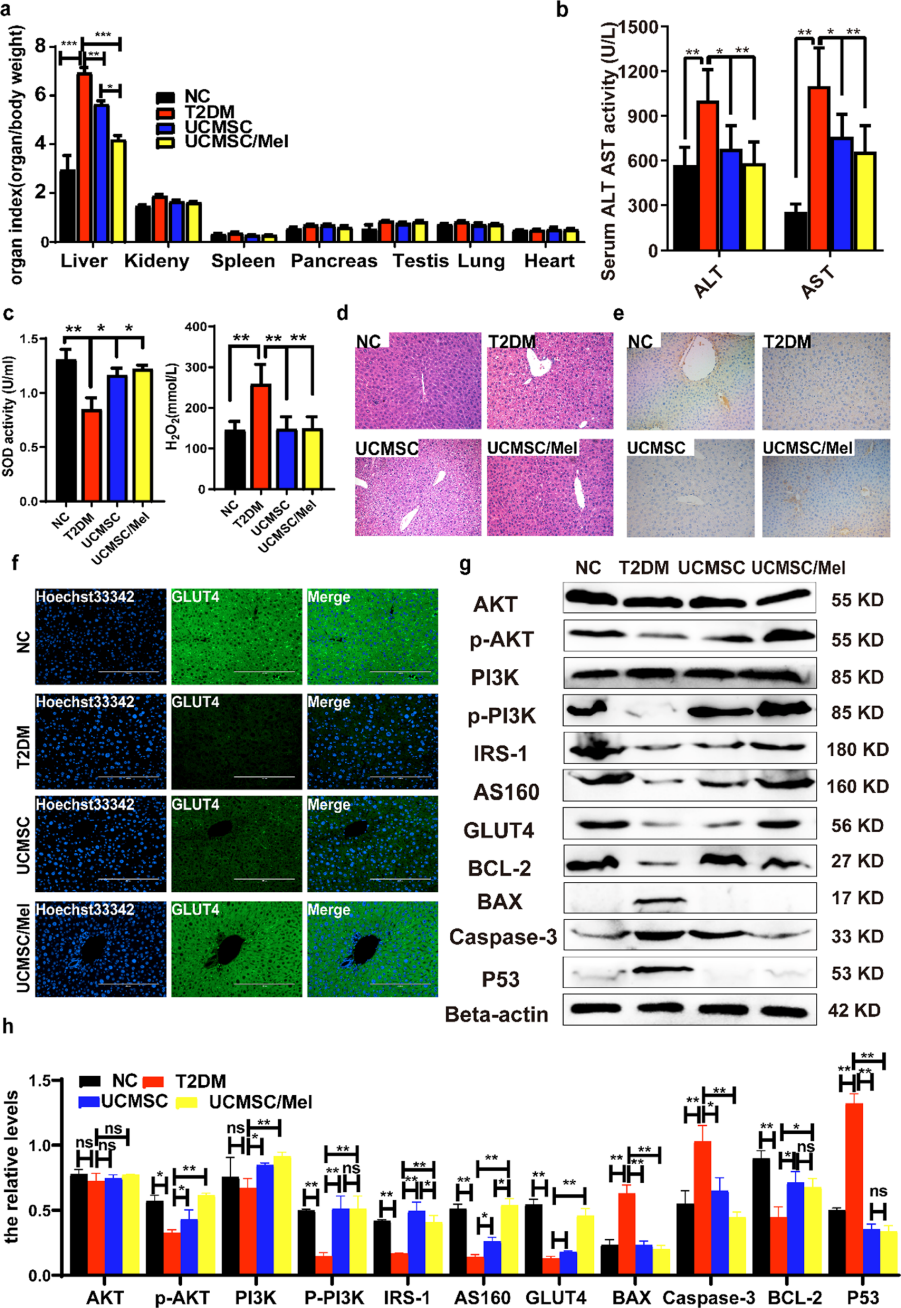
UCMSCs play a hypoglycemic role by regulating the activation of the PI3K/AKT signaling pathway. **a** Organ weight coefficient at 4 weeks after cell infusion. **b** Serum transaminase levels. **c** Hepatic level of SOD and H_2_O_2_. **d** H&E staining of liver tissue sections. Scale bar, 100 µm. **e** Immunohistochemical staining of IRS-1 in liver tissue. **f** Immunofluorescence staining of liver tissue GLUT4. **G** Western blot analysis of AKT, p-AKT, PI3K, p-P3K3, P53, GLUT4, Capase3, AS160, BAX, and BCL-2 expression levels in the livers of HFD-fed mice and T2DM, UCMSCs and UCMSCs/Mel after 6 weeks of treatment. Beta-actin was used as a loading control. **h** Quantitative analysis of the western blot results of G. NC (normal control); T2DM (Type II diabetic mellitus); UCMSC (hUC-MSCs); Mel (melatonin); ns (no significant). Data are mean ± SD; ^*^P < 0.05; ^**^P < 0.01; ^***^P < 0.001

To understand the mechanisms of UCMSC intervention for insulin resistance in a T2DM mouse model and to clarify whether UCMSC intervention is associated with the PI3K/Akt signaling pathway in hepatocytes, the liver is a vital place to regulate glucose metabolism and an important part of insulin action and catabolism. T2DM usually induces injury to liver histology and liver function. The organ coefficients (tissue weight/body weight) of mice in each group after 10 weeks are shown in Fig. 6a. We found significant differences in the index, and significant changes in the liver and pancreas between the type 2 diabetic and NC groups were observed. However, after four weeks of infusion with UCMSCs, the liver coefficients of the UCMSC and UCMSC/Mel groups were substantially lower than those of the type 2 diabetic group (Fig. 6a, *P* < 0.05 or *P* < 0.01). UCMSCs suppressed these trends, and the coefficients of related organs were restored to normal levels. There was no significance found in the lung and heart tissue coefficients of the different groups (Fig. 6a). The serum AST and ALT activities were significantly increased in the type 2 diabetic group compared with the NC group (*P* < 0.01). After administration of UCMSCs for four weeks, the serum levels of ALT and AST were notably lower than those in the type 2 diabetic group (Fig. 6b, *P* < 0.01). The activities of the antioxidant enzymes were evaluated. After UCMSC and UCMSC/Mel infusion, the activities of SOD significantly increased and the H_2_O_2_ content decreased compared to those in the type 2 diabetic group (*P* < 0.01 or *P* < 0.05), which showed that UCMSCs ameliorated liver injury (Fig. 6 c). There was less loose connective tissue and blebbing in the parenchymal cells (Fig. 6d). Moreover, the preventive and therapeutic treatment of UCMSC/Mel was more effective than UCMSC treatment. Liver damage was analyzed by H&E staining; glycogen storage is one of the vital functions of hepatocytes. The liver glycogen storage level, as a vital indicator of glucose metabolism in hepatocytes, was assessed via PAS staining (Additional file 1: Figure S5). The hepatic glycogen content in the NC and UCMSC/Mel groups was the highest compared to that in the T2DM and UCMSC groups. GLUT4 and IRS-1 play an important role in regulating glucose homeostasis in the whole body, and they were assessed by liver immunofluorescence and immunohistochemical staining (Fig. 6e, f). UCMSC/Mel infusion significantly increased IRS-1 and GLUT4 expression levels in type 2 diabetic mice. Western blot analysis showed that UCMSC and UCMSC/Mel infusion induced significant changes in the expression levels of cell apoptosis- and glucose metabolism-related genes (Fig. 6g, h, *P* < 0.01 or *P* < 0.05). This finding suggests that hUC-MSCs contribute to the activation of the PI3K/AKT signaling pathway.

## Discussion

Stem cell therapy has been widely used in clinical applications for degenerative diseases, ischemia-related organ dysfunction, diabetes mellitus, and promising treatment for a variety of neurological diseases. The ethical concerns and carcinogenic risks of embryonic stem cells (ESCs) and induced pluripotent stem cells (iPSCs) limited their general application in routine clinical treatment [20]. MSCs avoid these disadvantages and are an ideal seed cell type for cytotherapy. hUC-MSC therapies have the potential to differentiate into healed chronic diseases and are currently the most promising cells for diabetes mellitus therapy [21]. However, during in vitro culture, loss of stemness induced failure of MSC cytotherapy [12, 15] and thus became a critical problem hindering the wide application of MSC transplantation therapy. In this experiment, we developed a novel strategy based on a specific small molecule (melatonin) to preserve the stemness of hUC-MSCs. Treatment with low-dose melatonin relieves the senescence of MSCs and increases the proliferation and differentiation of MSCs [12, 22]. On the other hand, the combination of melatonin with MSCs in clinical application has not been reported. Therefore, the effect and safety of combined therapies with melatonin and stem cells for patients with T2DM-related insulin resistance, hyperglycemia and dysfunction remain uncertain. In this way, the present study was promising for diabetes therapy. Accordingly, our data encourage the need for a prospectively randomized clinical trial to investigate the efficacy and safety of such a combined regimen for patients with T2DM.

In this study, we investigated the therapeutic effects of melatonin treatment with hUC-MSCs in a mouse model of T2DM and yielded several specific implications. First, melatonin activated PI3K/AKT through the MT1 and MT2 receptor pathways, stimulated PDK1, PCNA and KI67, increased hUC-MSC proliferation and migration, and improved the efficacy of transplanted hUC-MSCs. Second, hUC-MSCs combined with melatonin therapy significantly improved cell therapy and the combined endpoint in the setting of HFD-induced hyperglycemia and insulin resistance syndrome. Third, the combined melatonin hUC-MSC regimen was superior to either alone in reducing pancreas parenchymal injury, inflammation, generation of oxidative stress, ROS, and apoptosis as well as augmenting the production of antioxidants. Third, combining hUC-MSCs and melatonin was superior to hUC-MSCs alone in repairing the damaged pancreatic islets and liver injury and generating oxidative stress, inflammation and apoptosis.

The pathophysiology of T2DM, also known as chronic disease and insulin-independent diabetes mellitus, is characterized by a state of persistent insulin resistance, pancreatic beta-cell dysfunction, abnormal incretin effect, hyperglycemia and excessive cell glucagon production are interrelated defects [22–25]. The prominent characteristic of T2DM is insulin resistance, which impairs the action of insulin. In the early stages of T2DM, enhanced secretion of insulin is indispensable to maintain blood glucose levels to overcome insulin resistance [26]. Nevertheless, with the progression of T2DM, insulin production fails to compensate for IR [27]. The crucial finding in the present study is that hUC-MSCs combined with melatonin therapy significantly increased the secretion of insulin and pancreatic islet repair. Furthermore, the combined melatonin hUC-MSC group regimen was superior to either single therapy in terms of morphological changes in the pancreatic islets, significantly increasing both pancreatic islet numbers and decreasing glucose expression levels. This was shown by the greater improvements in pancreatic β-cell function and insulin sensitivity compared with the hUC-MSC infusion group, suggesting that melatonin preserved the stemness of hUC-MSCs after being engrafted into the damaged tissue.

The liver is an important organ for lipid metabolism in the body and maintains the homeostasis of lipid synthesis and decomposition. However, HFD impairs this balance, leading to excessive accumulation of liver lipids (hepatic steatosis) and oxidative stress, indicating that liver damage has occurred [28]. Reactive oxygen species (ROS), such as superoxide anion (O_2_^−^), H_2_O_2_, and hydroxyl radical (HO-), consist of radical and nonradical oxygen species formed by the partial reduction of oxygen [29, 30]. Cellular ROS are generated endogenously in the process of mitochondrial oxidative phosphorylation, or they may arise from interactions with exogenous sources such as xenobiotic compounds [31]. When ROS overwhelm the cellular antioxidant defense system, whether through an increase in ROS levels or a decrease in the cellular antioxidant capacity, oxidative stress occurs. Oxidative stress results in direct or indirect ROS-mediated damage to nucleic acids, proteins, and lipids and has been implicated in insulin resistance, metabolic disorders, and diabetes [32]. There is an inherent defense mechanism, such as SOD and GSH, to protect cells from free radical attack. SOD can convert superoxide anion radicals into H_2_O_2_, which is catalyzed by calcium ions and glutathione peroxidase into water, which is the main source of water essential for the whole body [33]. Therefore, we selected SOD and H_2_O_2_ indices to evaluate the liver's ability to resist oxidative stress. Our study showed that UCMSC and UCMSC/Mel infusion significantly increased SOD levels in T2DM mice while decreasing H_2_O_2_ levels, indicating that UCMSC and UCMSC/Mel infusion may treat diabetes by relieving oxidative stress.

We further studied the ameliorating hyperglycemia mechanism of MSCs, which was primarily considered to be their potential to differentiate into β cells (insulin-producing cells), and a number of modified protocols have been used to increase their unlimited proliferation and differentiation efficiency [34–37]. We therefore tested insulin in the islets of T2DM mice that were injected with PKH26-labeled hUC-MSCs intravenously to detect whether any hUC-MSCs actively differentiated into β-cells. After careful 3D reconstruction analysis via observation under a fluorescence microscope, we did not find PKH26-labeled hUC-MSCs that colocalized in the pancreas islets. This means that in our T2DM model with only hUC-MSC injection, during the 7-day observation period, no transplanted hUC-MSCs were differentiated into β cells in the pancreas that produced insulin. This suggests that the beneficial effects of hUC-MSCs on the recovery of pancreatic islets may not involve their differentiation into β-like cells; rather, they improved serum insulin levels and anti-inflammatory factors as well as the production of growth and growth and antioxidant agents.

To unravel the mechanism of action of hUC-MSCs, we examined the insulin signaling cascade. Functional membrane translocation from the cytoplasm of glucose transporters in insulin-responsive tissues is cardinally regulated via the insulin signaling pathway. Elevation of the cascade phosphorylation of IRS-1/AKT/PI3K triggered via exogenous factors, including insulin, is essential for membrane translocation of GLUT4 [38, 39]. In the present study, we found that hUC-MSC infusion significantly regulates glucose transportation by activating AS160 on the insulin target cell membrane of T2DM mice, increases the expression level of GLUT4 and restores the high-fat diet-induced phosphorylation of IRS-1/PI3K/AKT. However, hUC-MSCs pretreated with melatonin infusion markedly upregulated p53-dependent BCL2 levels, inhibited the activation of BAX and Capase3, and decreased apoptosis. These findings suggest that the mechanisms of improved insulin sensitivity via hUC-MSC treatment may be caused, at least partially, by the enhanced effect of hUC-MSCs on insulin signaling transduction. Certainly, it cannot be excluded that hUC-MSC transplantation leading to enhanced expression levels of GLUT4 may, to some extent, compensate for the blunted insulin signaling pathway in T2DM mouse models. The precise mechanisms by which hUC-MSC transplantation regulates GLUT4 expression levels and IRS-1/PI3K/AKT phosphorylation remain to be determined.

## Conclusion

The results of the present study highlighted that hUC-MSCs pretreated with melatonin infusion were superior to either regimen of hUC-MSCs alone in ameliorating HFD- and STZ-induced T2DM in a mouse model through the downregulation of hyperglycemia insulin resistance, glucose tolerance, and oxidative stress, as well as the upregulation of serum insulin secretion and anti-inflammatory and antiapoptotic effects. These results indicated that hUC-MSCs controlled the levels of insulin tolerance and serum insulin in T2DM mice by affecting the expression of signaling factors in the PI3K/Akt signaling pathway. This study may provide important evidence for future clinical applications of combined melatonin hUC-MSC treatment as a novel T2DM therapy and cytotherapy.

## Supplementary Information

**Additional file 1**. Supplementary figures.

## Data Availability

All data generated and analyzed during this study are included in this published article and supplementary information file.

## Abbreviations

AS160: A 160 kDa substrate of the Akt Ser/Thr kinase
ADMSCs: Adipose-derived mesenchymal stem cells
ALT: Alanine aminotransferase
AST: Aspartate aminotransferase
BCL-2: B-cell lymphoma-2
BAX: B-cell lymphoma-2-associated X
Caspase3: Cysteine protease protein
ELISA: Enzyme-linked immunosorbent assay
GO: Gene Ontology
GLUT4: Glucose transporter type 4
GAPDH: Glyceraldehyde phosphate dehydrogenase
HE: Hematoxylin and eosin
hUC-MSCs: Human umbilical cord mesenchymal stem cells
H_2_O_2_: Hydrogen peroxide
IRS-1: Insulin receptor substrate 1
IPITT: Intraperitoneal insulin tolerance tests
KEGG: Kyoto Encyclopedia of Genes and Genomes
Mel: Melatonin
MT1: Melatonin receptor 1
MT2: Melatonin receptor 2
NC: Normal control
OGTT: Oral glucose tolerance tests
PAS: Periodic acid Schiff
PI3K: Phosphoinositide 3-Kinase
AKT: Protein kinase B
PDK1: Phosphoinositide-dependent protein kinase 1
PCNA: Proliferating cell nuclear antigen
RIPA: Radio-immunoprecipitation assay
SOD: Superoxide dismutase
TC: Total cholesterol
TG: Triglyceride
