# Supplementary material for: Melatonin treatment improves human umbilical cord mesenchymal stem cell therapy in a mouse model of type II diabetes mellitus via the PI3K/AKT signaling pathway
Source: Stem Cell Res Ther. 2022 Apr 12;13:164. doi: 10.1186/s13287-022-02832-0 (PMC9006413; doi:10.1186/s13287-022-02832-0)
Supplement: Supplementary file 1 — Additional file 1. Supplementary figures. [file 13287_2022_2832_MOESM1_ESM.docx]

**Supplementary figures and figure legends**

**
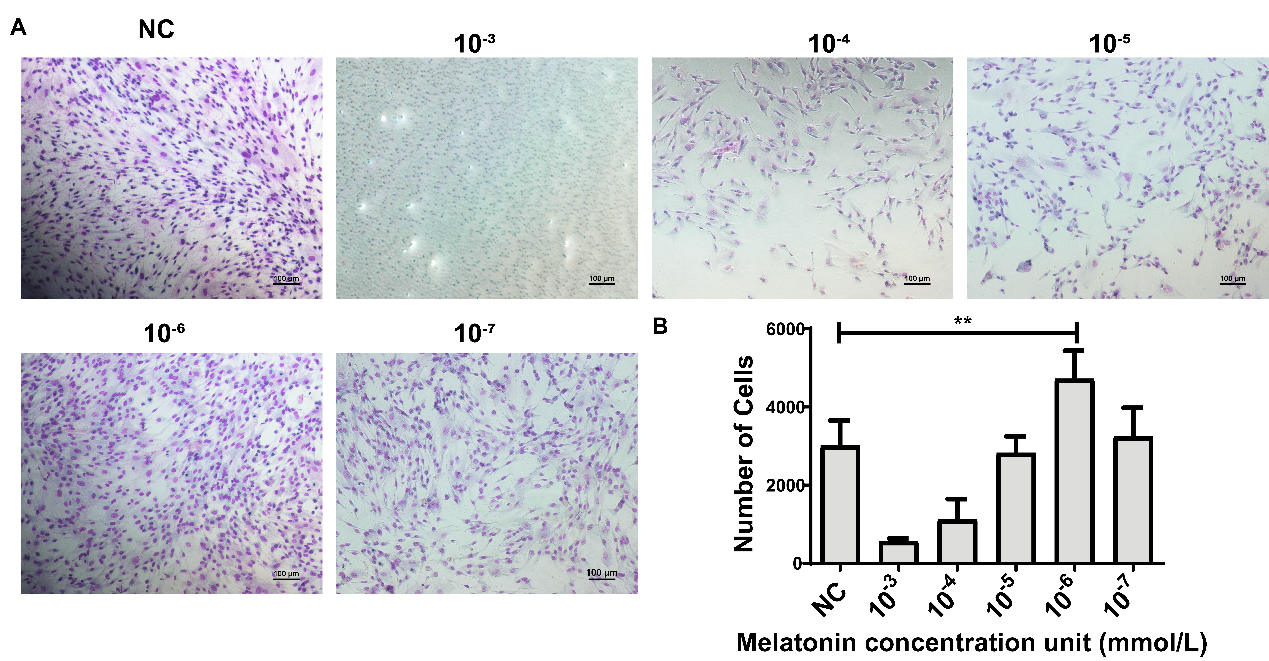
**

**Supplementary Figure 1. Investigate the effects of melatonin concentration on the proliferation hUC-MSCs**

The cells were treated with melatonin at 1*10^-3^ mmol/L and 1*10^-7^ mmol/L concentration. The Gemsa staining results showed that treatment with 1*10^-6^ mmol/L melatonin improved cell proliferation of hUC-MSC. Values are the mean ± SEM of six independent experiments (n = 6) in Gemsa staining. Statistically significant differences are indicated by *where *p* < 0.05 or **where *p* < 0.01 between the indicated groups.

**
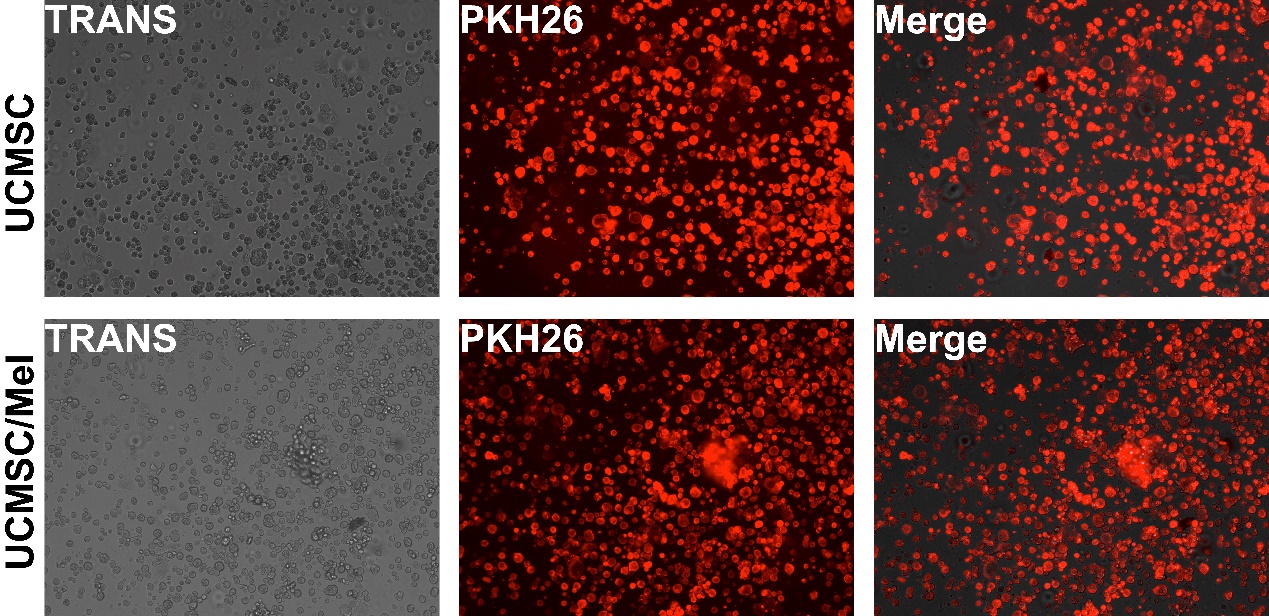
**

**Supplementary Figure 2. Labeling hUC-MSCs with PKH26 (red)**

The PKH26 red fluorescent cell linker kit was used to label the hUC-MSCs before transplantation.


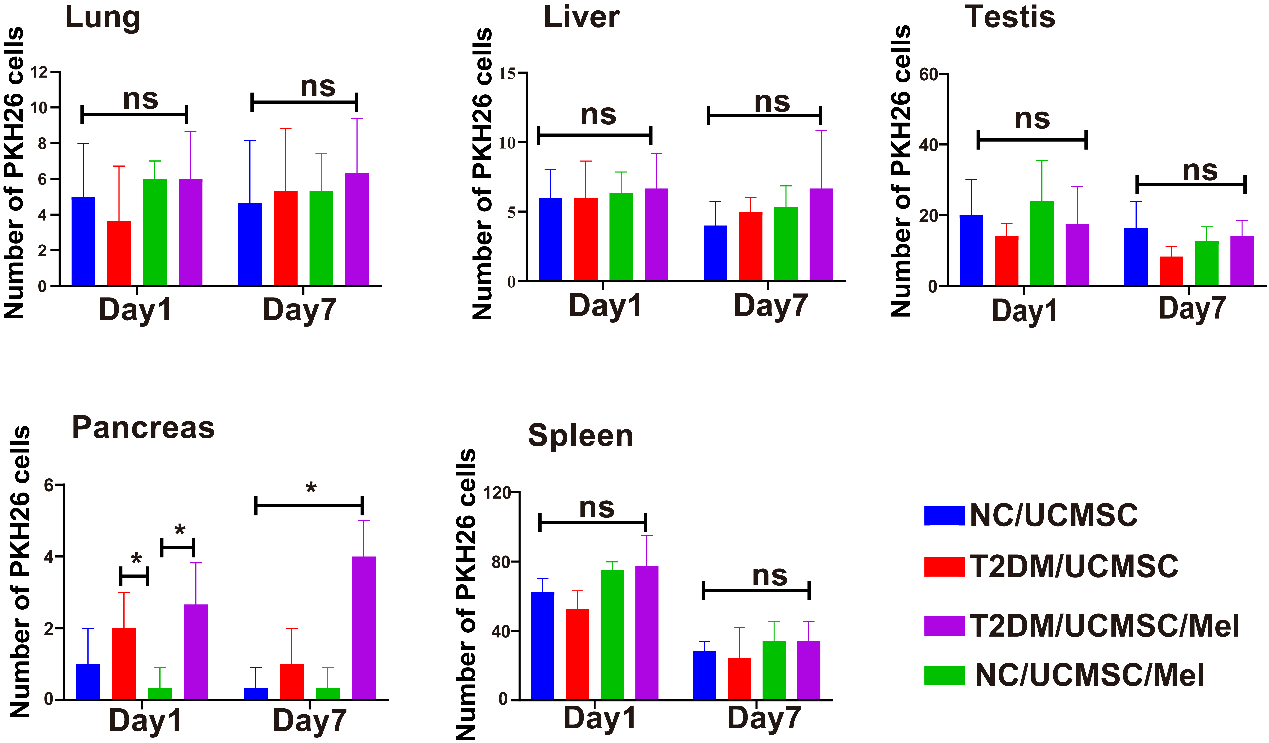


**Supplementary Figure 3. The quantitative analysis of the Colonization of UCMSC/Mel in the recipient mice organ.**

NC (normal control); T2DM (Type II diabetic mellitus); UCMSC (hUC-MSCs); Mel (melatonin). Data are mean ±SD of (n=8); ^*^*P* < 0.05; ^**^*P* < 0.01; ^***^*P* < 0.001.


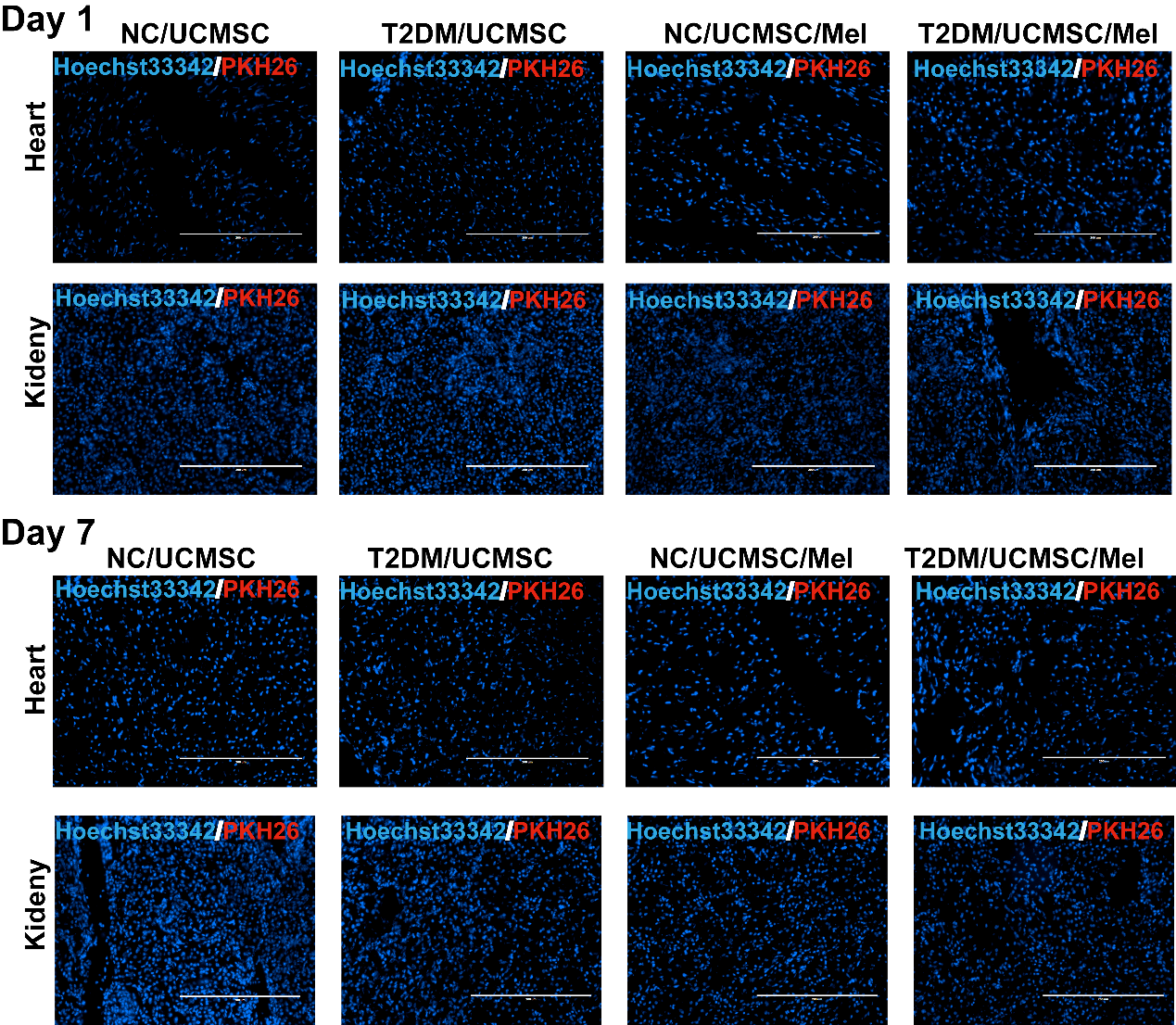


**Supplementary Figure 4. Colonization of UCMSC/Mel in the recipient mice organ.**

Engraftment of UCMSCs and UCMSC/Mel was undetected in the heart and kidney of T2DM mice among the organs examined. UCMSCs were PKH26 (red) labelled in advance. After the infusion, recipients were sacrificed on day 1, and day 7, and UCMSCs tracking in heart and kidney were evaluated using a confocal laser scanning microscope.


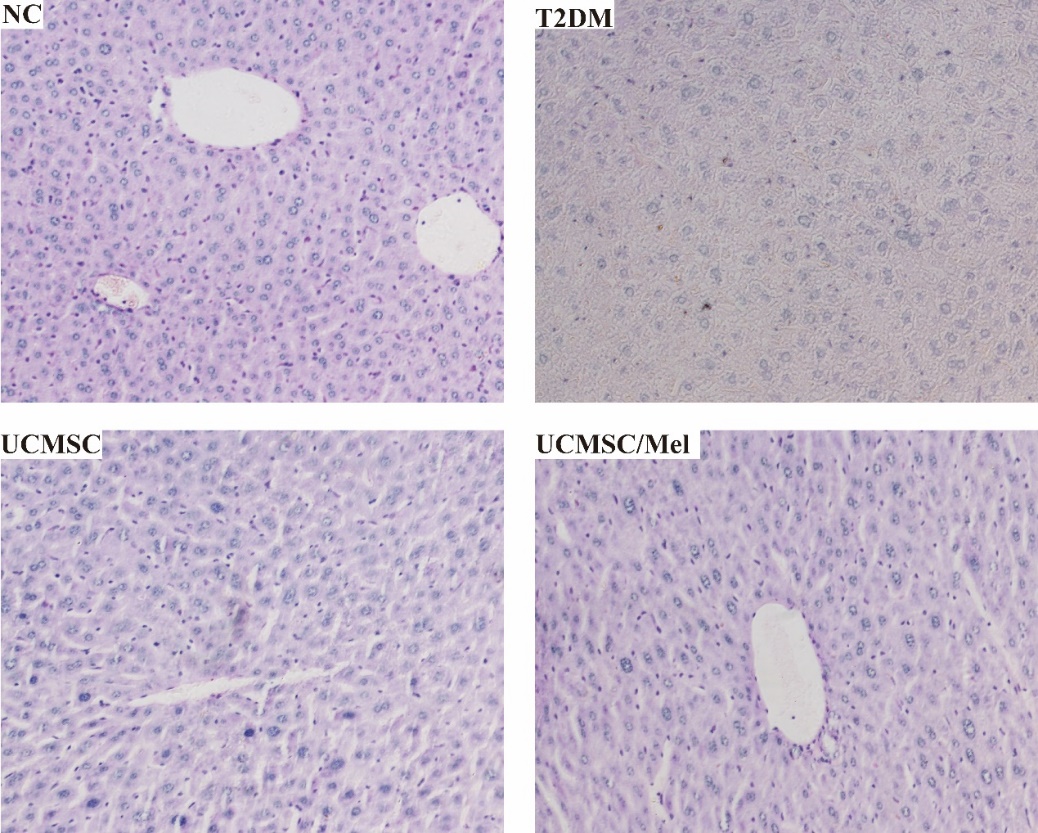


**Supplementary Figure 5. Histopathological features after PAS staining of liver sections from a representative T2DM mice of each group**

Demonstrated that in NC and UCMSC/Mel Groups liver tissue the quantity of glycogen was the highest as compared to T2DM and UCMSC group. Scale bars, 100 μm. NC (normal control); T2DM (Type II diabetic mellitus); UCMSC (hUC-MSCs); Mel (melatonin).
